# LDH-Based “Smart” Films for Corrosion Sensing and Protection

**DOI:** 10.3390/ma16093483

**Published:** 2023-04-30

**Authors:** Xuejie Zhao, Yujie Yuan, Yuankun Wei, Zhe Zhang, You Zhang

**Affiliations:** 1 College of New Materials and Chemical Engineering, Beijing Institute of Petrochemical Technology, Beijing 102617, China; 2 Beijing Key Lab of Special Elastomeric Composite Materials, Beijing 102617, China

**Keywords:** layered double hydroxide, 8-hydroxyquinoline, films, corrosion sensing, corrosion inhibition, aluminum alloy

## Abstract

In a “smart” corrosion-protective coating system, both the active anti-corrosion and the early corrosion detection of underlying metals are highly required. It is practical significant to develop materials that possess self-detecting of the early local corrosion and self-healing of coating defects simultaneously. The organic compound 8-hydroxyquinoline (8HQ) is an effective inhibitor and a fluorescent sensor probe for corrosion of aluminum alloy. Therefore, a layer double hydroxide (LDH) nanocontainer film loaded with the 8HQ was developed for the active corrosion protection purpose of aluminum alloy AA2024. In corrosive environments, the 8HQ are released from LDH film to inhibit the corrosion process, leading to the loss of the complexation with Al^3+^ ions in LDH laminates, thus turning off fluorescence. Results show that the LDH film loaded with 8HQ composites can improve the anti-corrosion performance of the film by releasing corrosion inhibitors on demand. Simultaneously, due to the complexation of 8HQ and Al^3+^ ions, the LDH film is fluorescent at the initial stage under ultraviolet light, and then becomes non-fluorescent at the corrosion sites, indicating the corrosion evolution process of the coating. The 8HQ-loaded LDH film with self-healing and self-detecting dual functions provides promising opportunities for the effective corrosion protection of aluminum alloy due to its “smart” and multifunctional properties.

## 1. Introduction

Aluminum alloy AA2024 is widely used in the aviation industry due to its good mechanical properties [1]. Nevertheless, AA2024 often appears pitting corrosion or intergranular corrosion by the existence of intermetallic compounds, which affects the service process [2]. For a long time, the protection of aluminum alloy depended on the traditional chromate conversion coating [3]. However, on account of the serious harm of chromate toxicity to the human body and the environment, chromate conversion technology has been gradually banned [4]. Therefore, the development of a chromium-free treatment has become a hotspot in the research of aluminum alloy corrosion protection [5].

Layered double hydroxide (LDH), as a new surface treatment technique of aluminum alloy, has the potential to replace chromate conversion coating [6]. The chemical formula of the LDH can be expressed as [M^2+^_1−x_M^3+^_x_(OH)_2_]^x+^A^n−^_x/n_·mH_2_O. M^2+^ and M^3+^ are divalent and trivalent metal cations, respectively, which occupy octahedral pores of the brucite-like hydroxides and form a metal hydroxides layer. A^n−^ is an anion which occupies the inter-layer of the metal hydroxides layer [7]. LDH has the unique advantage of anion exchange and can be replaced by other anions in the preparation process, making it an effective nano-storage for corrosion inhibitors [8]. In the process of corrosion prevention, the corrosion inhibitor anions loaded in the LDH inter-layer are stimulated and released by the external corrosion media, and the corrosive anions can be captured at the same time [9] to enhance the corrosion resistance [10,11] and self-healing performance [12,13] of the substrate.

However, the self-healing ability of the LDH film can diminish over time due to the erosion caused by harsh conditions. This can significantly weaken the protective ability of the coatings, ultimately leading to the corrosion of the metal substrates [14]. Generally, the initial stage of corrosion is invisible to the naked eye. Visibility to the naked eye means that corrosion has already developed and expanded [15]. Therefore, before obvious corrosion occurs, autonomous warning by early corrosion detection is of great significance for extending the life-time of coatings and metal substrates. Common sensing species in self-detecting coatings include color indicators (1,10-phenanthroline [16], phenolphthalein [17]) and fluorescent indicators (8-hydroxyquinoline [18], coumarin [19]). The coloration signal may be negatively influenced by either the color of the coating or the corrosion products [20]. The occurrence of localized corrosion of metals is accompanied by the production of metal ions and the acidification of the immediate environment [21]. Theoretically, the detection of local corrosion can be achieved through changes in fluorescence behavior resulting from the influence of metal ions and hydrogen ions [22]. The ideal fluorescent indicator should also respond to local pH or metal ions changes associated with corrosion resulting in “turn-on” fluorescence or “turn-off” fluorescence.

The organic compound 8-hydroxyquinoline (8HQ) is a class of common photosensitive compound, which has a good affinity for aluminum. It can be combined with Al^3+^ to form 8-hydroxyquinoline aluminum (AlQ_3_), which can emit a fluorescence of a certain wavelength and intensity under ultraviolet irradiation [23]. Therefore, 8HQ and its derivatives have been used as fluorescent corrosion indicators. An investigation was conducted on a coating containing a complex (AlQ_3_) of Al^3+^ ions with 8HQ, to develop a fluorescent technology for predicting corrosion in aluminum alloy [23]. Fluorescent spots were clearly visible under UV light after conducting salt spray tests, namely corrosion pitting under an optical microscope, whereas there was no clear change under visible light. The corrosion process of aluminum alloy AA6061 can be well monitored by using 8HQ as photosensitive compound and corrosion weightlessness method [24]. Meanwhile, it was reported that 8HQ were dispersed into the inter-layer region of hydrotalcite-like compound using anion exchange method, in which Al^3+^ coordinated with 8HQ. This hydrotalcite-like compound has the potential to serve as a novel luminescent functional material [25].

In addition, 8HQ is famous for its excellent bonding and chelating behaviors. The strong coordination of 8HQ and its derivatives with metal surfaces results in the formation of various coordination complexes, which in turn enhances their inhibition behaviors. At present, 8HQ and its derivatives are applied to effective and green corrosion inhibitors for steel, copper, aluminum, and magnesium alloys [26]. In 3.5 wt.% NaCl solution, 8HQ inhibitor molecules were adsorbed on a 7075 aluminum alloy surface, resulting in an increase in inhibition efficiency up to 96%. It was confirmed that 8HQ could be used as an effective corrosion inhibitor for aluminum alloy in 3.5 wt.% NaCl solution [27]. As a type of mixed inhibitor, 8HQ reduces the rate of both anodic and cathodic reactions by creating an insoluble chelate Al(HQ)_3_ on AA2024-T3 surface to hinder the adsorption of chloride ions [28]. The intercalation of 8HQ anions has no obvious effect on the microstructure of MgAl hydrotalcite coating on aluminum substrate, but confers the coating long-term corrosion resistance [29]. A successful fabrication of 8HQ intercalated Mg-Al base LDH coating on AZ31 was achieved. Due to the ability of anion exchange and chelation to form Mg(HQ)_2_ redeposition, magnesium alloy exhibits good long-term corrosion protection and self-healing properties [30]. Similar conclusions have been strongly confirmed by many other studies [31,32,33].

This study demonstrates a novel approach to achieving simultaneous corrosion sensing and protection by developing a composite film that is loaded with 8HQ (corrosion inhibitor and fluorescent indicator) and synthesized using a two-step process (the hydrothermal process and the anion exchange process). The intercalation of 8HQ into LDH enables fluorescence emission when complexing with Al^3+^ in the laminates. Furthermore, the release of 8HQ from the LDH film by anion exchange with Cl^−^ results in fluorescence quenching when there is no interaction with Al^3+^. This process enables the early detection of corrosion and marks a significant advancement in the field of corrosion sensing. Additionally, the use of 8HQ as an organic corrosion inhibitor for aluminum alloy highlights the potential for this material to provide both sensing and protection functions. Overall, the development of this composite film represents an innovative and promising approach to addressing the challenges associated with corrosion detection and prevention.

## 2. Experimental Section

### 2.1. Materials

In this work, the aluminum alloy AA2024 plates (mean composition in wt.% of Cu 3.8, Mg 1.3, Mn 0.43, Cr 0.1, Zn 0.23, Ti 0.1, and balanced Al) with a size of 25 mm × 25 mm × 5 mm were ground and polished to 1200 grit with SiC sandpapers. The samples were rinsed in deionized water, cleaned by ethanol (>98%), and then dried in cold air.

### 2.2. Synthesis of the LDH Films

The mixture solution of 0.05 M Zn(NO_3_)_2_·6H_2_O (99 wt.%, Aladdin, Shanghai, China) and 0.3 M NaNO_3_ (AR, Sinopharm Chemical Reagent Co., Ltd., Shanghai, China) was prepared by adjustment of the pH to 6.3 with 1% ammonia. The cleaned AA2024 plates were vertically immersed in the above solution at 75 °C for 24 h. After that, the samples were washed with deionized water and ethanol, and then dried in air. ZnAl-NO_3_-LDH films were prepared on the surface of the samples by an in-situ growth approach (named LDH-NO_3_).

The 8HQ corrosion inhibitor anions were intercalated into the LDH inter-layer gallery by anion exchange reaction. An amount of 1.0 g NaOH (99.9%, Beijing Chemical Works, Beijing, China) was added to 25 mL of ultrapure water, followed by the dissolution of 0.2 g 8HQ (C_9_H_7_NO, 99.5%, Tianjin Guangfu Fine Chemical Research Institute, Tianjin, China) by vigorous stirring. ZnAl-8HQ-LDH films were obtained by the immersion of ZnAl-NO_3_-LDH samples in the above 8HQ solution at 50 ℃ for various times (x = 5 min, 30 min, 1 h, and 6 h). After immersion, the fabricated films were rinsed with deionized water and ethanol, then dried in air (named LDH-8HQ).

### 2.3. Morphology and Structure Analysis

The optical macro images of the LDH films were recorded by a digital camera. The microcosmic surfaces and cross-sectional morphologies of the LDH films were detected by a field emission scanning electron microscope (FE-SEM, JEOL JSM-7800) at 10 kV accelerating voltage. Energy dispersive spectrum (EDS, Oxford Instrument Isis 300) was utilized to study the element content and the elemental distribution. All the samples were sputtered with gold to increase conductivity. The crystalline structures of the LDH films were identified using X-ray diffraction (XRD) with Cu Kα1 radiation (λ = 0.154 nm) in a 2θ angle ranging from 3 to 75°. The different types of chemical bonds of the samples were characterized by a Fourier transform infrared spectrometer (FTIR, Bruker Tensor 27 OPUS) over a wavenumber range of 400–4000 cm^−1^.

### 2.4. Corrosion Characterization

The corrosion behavior of the films was evaluated using electrochemical impedance spectroscopy (EIS) and potentiodynamic polarization curves. The experimental setup (Wuhan Corrtest CS350 electrochemical workstation) consisted of a three-electrode system, where the working electrode was the sample with an exposed area of 1 cm^2^, the counter electrode was a platinum plate, and the reference electrode was an Ag/AgCl electrode. The tests were conducted in a 3.5 wt.% NaCl solution at room temperature, after stabilizing the open circuit potential (OCP) of the working electrode in the electrolyte. During the experiment, a sine wave with an amplitude of ± 10 mV and a frequency range of 10^−2^–10^4^ Hz was used. An equivalent circuit was used to model the impedance spectrum data obtained during the process. The Tafel dynamic potential polarization test was conducted with a scanning speed of 1 mV·s^−1^, and the scanning range was from −0.25 V to + 0.5 V relative to the OCP.

### 2.5. Fluorescence Performance Test

The fluorescence performance test was studied using XS-600M3G fluorescence microscope to monitor the change of fluorescence intensity of the LDH films. The fluorescence intensity of the samples after immersion in 3.5 wt.% NaCl solution for different days was analyzed, and then the fluorescence self-detecting mechanism was revealed.

## 3. Results and Discussion

### 3.1. Effect of Anion Exchange Reaction Time on the Morphology and Structure of the LDH Films

**Figure 1:**
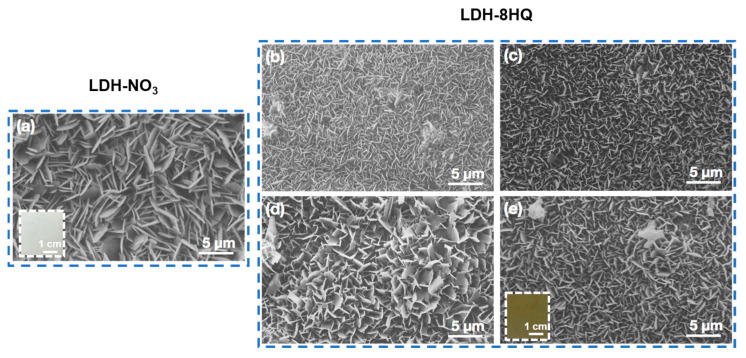
Surface SEM images of (**a**) the LDH-NO_3_ film and the LDH-8HQ films with different anion exchange reaction times: (**b**) 5 min, (**c**) 30 min, (**d**) 1 h, (**e**) 6 h.

The optical photographs and SEM surface micrographs of the LDH films are presented in Figure 1. The LDH-NO_3_ film in Figure 1a is composed of typical vertically-grown nanosheets [34], and the cross-linked hexagonal nanosheets almost cover the whole substrate surface, which is in accordance with our previous studies [11,35,36]. Figure 1b–e show the LDH-8HQ films with different anion exchange reaction times. No obvious changes in morphology and structure can be observed as compared to that without an anion exchange reaction [31]. It can be seen that after the intercalation with 8HQ, the color of the modified film seems to evenly change from white to yellow [29], as shown in the insets in Figure 1a,e. With the loading time of 8HQ, the nanosheets are thicker and the surface is more uniform and dense, indicating that the growth of the LDH film loaded with 8HQ for 6 h is better, which may further enhance the corrosion resistance of the LDH film.

**Figure 2:**
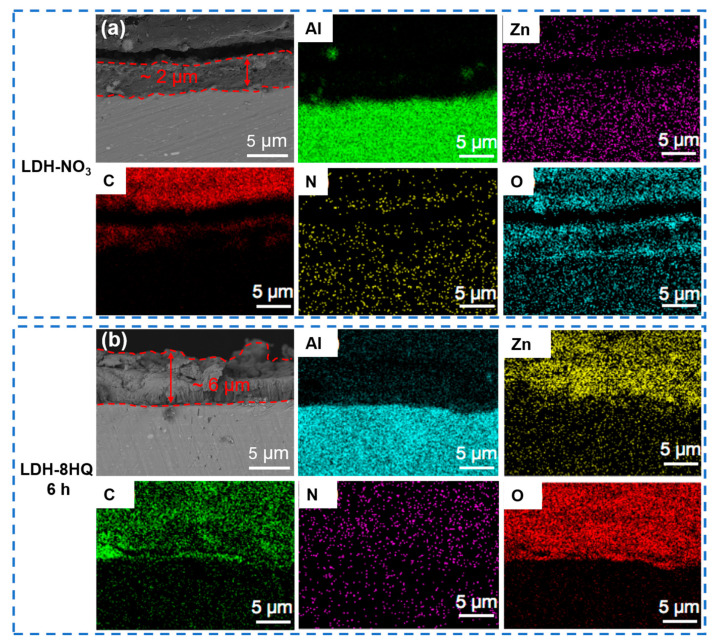
Cross-section SEM images and corresponding EDS mapping of (**a**) the LDH-NO_3_ film and (**b**) the LDH film loaded with 8HQ for 6 h.

In order to further study the elemental composition and distribution of the LDH films, Figure 2 shows the cross-section morphologies and corresponding EDS mapping results of the LDH films before and after loading the 8HQ corrosion inhibitor. The samples were encapsulated and fixed with epoxy resin to facilitate SEM observation [37]. A large number of Al, Zn, N, and O elements are distributed in the LDH-NO_3_ film in Figure 2a, among which the Al element comes from the AA2024 matrix, and the Zn, N, and O elements mainly come from the LDH-NO_3_ film, suggesting that the LDH-NO_3_ film is densely grown and tightly bonded on the surface of the matrix [38]. In addition, the thickness and structure of the LDH films can be qualitatively analyzed through the cross-section morphologies and corresponding EDS mapping results [39,40,41]. The thickness of the LDH-NO_3_ film is roughly 2 μm, and after anion exchange reaction loading 8HQ for 6 h, the thickness changes significantly to about 6 μm. It is worth noting that the C element primarily from 8HQ is concentrated on the LDH-8HQ film in Figure 2b, which signifies the existence of 8HQ in the LDH film through anion exchange process. The LDH-8HQ film grows uniformly and vertically from the bottom layer and has strong adhesion with the substrate. The thickness of the LDH film loaded with NO_3_^−^ to 8HQ anions is gradually increased, which is more conducive to blocking the penetration of aggressive ions into aluminum alloy.

**Figure 3:**
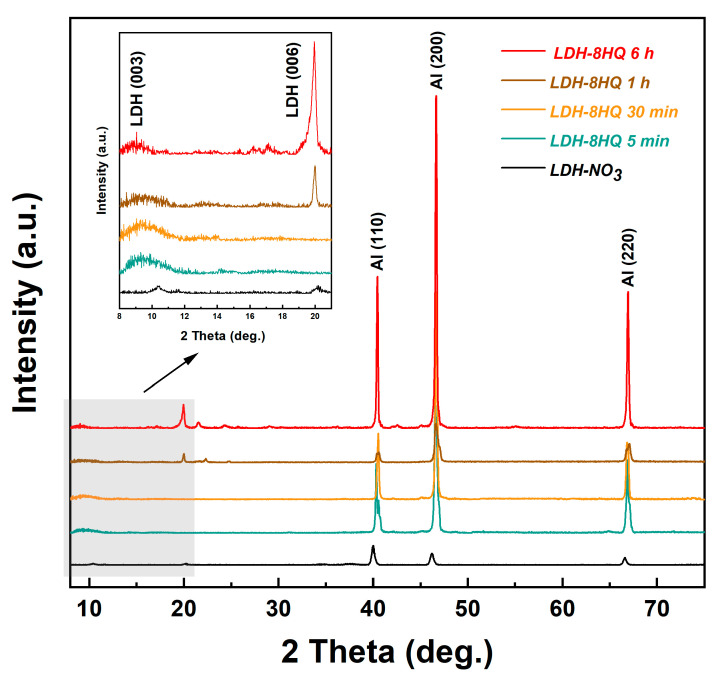
XRD patterns of the LDH-NO_3_ film and the LDH-8HQ films loaded with 8HQ for different times.

Figure 3 presents the XRD patterns of the LDH-NO_3_ film and the LDH-8HQ films loaded with 8HQ for different times. Two major characteristic diffraction peaks of the LDH-NO_3_ film show that the reflection intensity of (006) and (003) decrease. It is speculated that a certain angle of reflection is required for the XRD detection of the LDH film, which indicates that the LDH film is grown perpendicular to the metal substrate and has the definite orientation [42,43,44]. On the other hand, the observed low (003) reflection intensity may be due to the high atomic scattering factor of M-Al-hydroxide (the “host” layer) [45,46], which may be caused by the loss of crystallinity [44,47]. From the partial enlargement in Figure 3, with the progress of anion exchange reaction, the diffraction reflection (003) moves to lower angles, which confirms that 8HQ inhibitor anions are successfully intercalated into the LDH galleries [30,31,34]. The characteristic peaks of the LDH-8HQ film are more evident after loading 8HQ for 6 h. Based on the analysis of surface and cross-section SEM, it is confirmed that the LDH-8HQ film grown for 6 h has a dense morphology and good crystal structure.

**Figure 4:**
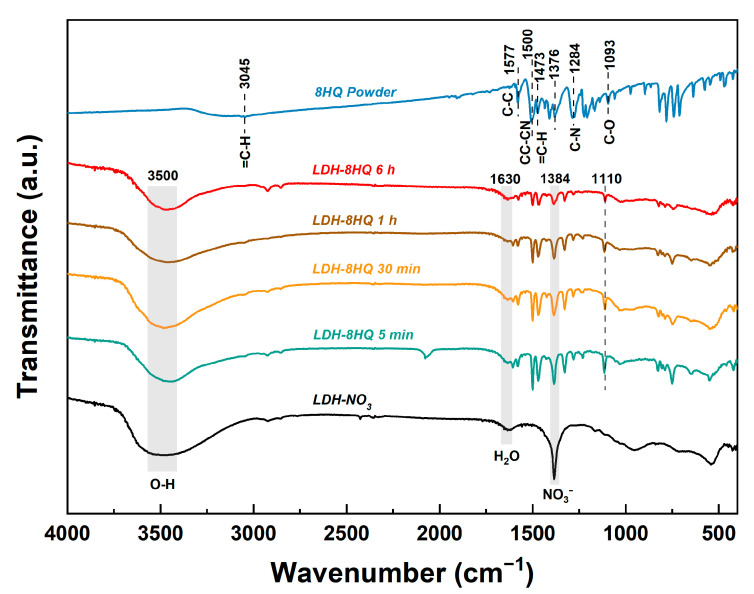
FTIR spectra of the LDH-NO_3_ film, the LDH-8HQ films loaded with 8HQ for different times, and the 8HQ powder.

The FTIR spectra of the LDH films before and after modification with 8HQ are recorded in Figure 4 for more insights into the chemical bonding. In addition, the FTIR spectra of the 8HQ powder are studied for reference. The spectra of all the films exhibit characteristic absorption peaks that are commonly associated with LDH. A prominent and broad absorption band located at approximately 3500 cm^−1^ can be attributed to the hydroxyl stretching vibration *m* (OHstr), which arises from hydroxyl groups in the hydrotalcite-like layer as well as water molecules in the inter-layer [10,35,36]. Furthermore, another absorption band observed at around 1630 cm^−1^ results from the hydroxyl bending vibration *δ* (H_2_O) of water molecules [25,35]. The absorption band recorded at 1384 cm^−1^ corresponds to the stretching vibration of inter-layer NO_3_^−^ [39,48]. Compared with the LDH-NO_3_ film, the NO_3_^−^ characteristic absorption band weakens after intercalation with 8HQ, which is likely due to the occurrence of anion exchange reaction [8]. At low wavenumber range (400–700 cm^−1^), the bands are primarily related to M-O, M-O-M, and O-M-O bond vibrations [30,43,49]. The obtained results provide strong evidence for the successful preparation of the LDH-NO_3_ film.

The spectra of the LDH-8HQ films loaded with 8HQ for different times are roughly similar to those of the 8HQ powder. The vibration peaks at 1577 cm^−1^ and 1376 cm^−1^ in the spectra are ascribed to the C-C stretching [30,43], while the peak at 1500 cm^−1^ is attributed to the CC-CN stretching vibration within the quinoline ring [49,50]. The peaks observed at 1473 cm^−1^ and 3045 cm^−1^ are respectively derived from the C-H bending and stretching vibrations of the 8HQ ring system [43]. Additionally, the absorption peak at 1284 cm^−1^ is associated with the C-N stretching vibration within the quinoline ring [49]. It is noteworthy that the C-O stretching vibration peak of the 8HQ shifts from 1093 cm^−1^ to 1110 cm^−1^ (the LDH-8HQ films), which suggests the formation of C-O-M coordination bonds between some of the C-O bonds in the 8HQ and the Al^3+^ ions present in the LDH laminates [25,49,50]. The SEM, XRD, and FTIR analysis collectively demonstrate the successful intercalation of 8HQ, resulting in the formation of LDH coating on AA2024 substrate.

### 3.2. Effect of Anion Exchange Reaction Time on the Corrosion Resistance of the LDH Films

**Figure 5:**
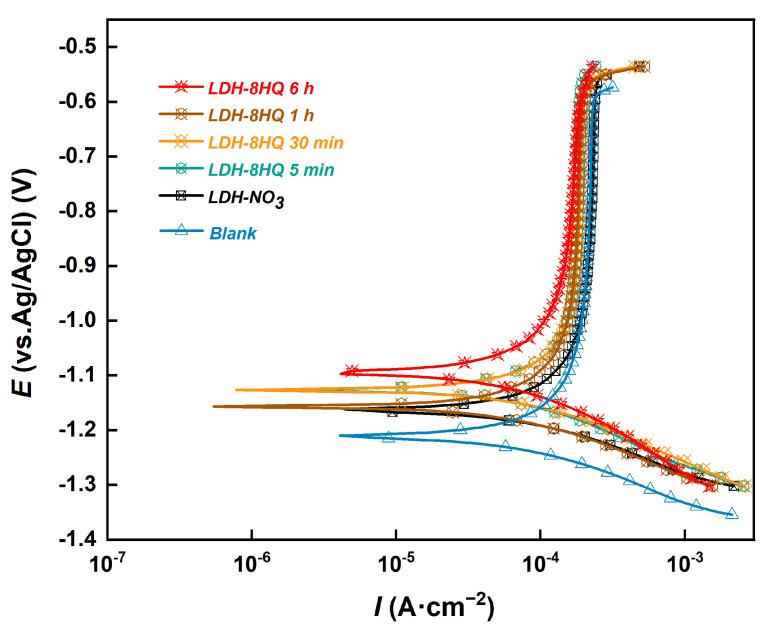
Potentiodynamic polarization curves of the samples immersed in 3.5 wt.% NaCl solution.

To assess the corrosion resistance of the obtained LDH films, the potentiodynamic polarization measurement was conducted in 3.5 wt.% NaCl solution. The resulting potentiodynamic polarization curves are exhibited in Figure 5. The electrochemical parameters are determined using the Tafel linear extrapolation method [51]. These parameters include corrosion potential (*E*_corr_), corrosion current density (*I*_corr_), anodic slope (*b*_a_), cathodic slope (*b*_c_), and polarization resistance (*R*_p_), which are illustrated in Table 1. A lower value of *I*_corr_ indicates the higher corrosion resistance [52,53]. The Stern–Gary equation (Equation (1)) was used to calculate the polarization resistance (*R*_p_) [32]:

(1) $$R_{p}=\frac{b_{a}\;\times \;b_{c}}{2.303\;\times \;I_{\mathrm{corr}}\left(b_{a}+b_{c}\right)}$$

It can be clearly seen that the *E*_corr_ of the ordinary LDH-NO_3_ film is the most negative (−1.161 V) and the *I*_corr_ is the highest (8.945 × 10^−4^ A·cm^−2^) among all the coating samples, indicating that the corrosion resistance of the LDH film is improved after loading 8HQ. The polarization curves of 8HQ-loaded LDH films prepared with different anion exchange reaction times for 5 min, 30 min, 1 h, and 6 h are observed in Figure 5. The *I*_corr_ decreases significantly and the *E*_corr_ shifts toward the positive direction with time, which indicates that the LDH film loaded with 8HQ for 6 h provides better corrosion resistance in comparison with other times. Moreover, the higher the *R*_p_ value is, the better the corrosion resistance has [32,54]. The inhibition efficiency (IE or *η*) can be calculated through *R*_p_ as (Equation (2)):

(2) $$\eta =\frac{{R\;}_{p}^{\mathrm{inh}}-{R\;}_{p}^{\mathrm{blank}}}{{R\;}_{p}^{\mathrm{inh}}}$$

where $R_{p}^{\mathrm{inh}}$ is the average polarization resistance of the inhibited sample and $R_{p}^{\mathrm{blank}}$ is the average value of the blank non-inhibited sample [55]. After being loaded with 8HQ for 6 h, the LDH–8HQ film demonstrates a significant improvement of 93.9% in inhibition efficiency compared to the blank sample. These findings are consistent with the SEM observation, which indicates that the LDH film structure is well-formed after 6 h of loading 8HQ and can serve as an effective barrier against the penetration of aggressive electrolytes.

**Figure 6:**
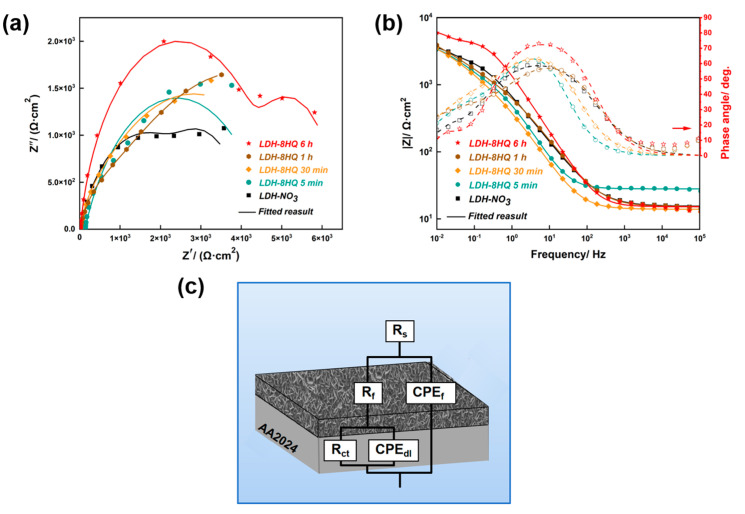
(**a**) Nyquist plots, (**b**) Bode plots and (**c**) Equivalent electric circuit of the samples immersed in 3.5 wt.% NaCl solution.

As a nondestructive technique, the EIS measurement was performed to estimate the corrosion resistance of the samples in 3.5 wt.% NaCl solution [56]. The EIS curves, both the Nyquist and the Bode impedance plots, can effectively reflect the LDH films corrosion performance in Figure 6a,b, respectively.

The anti-corrosion performance of the LDH films can be characterized by the capacitive circuit radius in the Nyquist plot in Figure 6a [57]. The charge transfer process manifests the characteristics of the double electric layer at the interface between the electrolyte and the electrode, and the charge transfer resistance causes the capacitive circuit [54,58]. The capacitive circuit radius of the LDH-8HQ film loaded for 6 h is the largest, followed by other loading times. This evidence indicates that the LDH film loaded with 8HQ for 6 h has the best protective performance, which reveals the permeability of the corrosion electrolyte into the substrate is lower than that of other films, thus reducing the corrosion rate of the substrate. The observed results are consistent with the polarization curves in Figure 5.

In general, the overall anti-corrosion performance of the LDH films can be revealed by the low frequency impedance modulus (|Z| at 0.01 Hz) in the Bode plots of the impedance modulus in Figure 6b [11,56,57]. The 6 h of 8HQ-loaded LDH film displays the highest |Z| value compared with that of the other films, which further demonstrates that it has a superior protection for AA2024. Two time constants can be found in the Bode plots of the phase angle in Figure 6b, which occur in the medium and low frequency regions. The time constant is corresponding to the presence of thin oxide film between the AA2024 substrate and the LDH layer. Moreover, another time constant is in regard to the LDH film response at the interface of corrosive solution [11,59]. With the time of loading the 8HQ inhibitor, the second time constant related to the Bode phase angle plot is extended over a wide frequency range (10^−1^–10^3^ Hz). The frequency expansion of the phase angle is the largest for 6 h of the 8HQ-loaded LDH film compared with other times [58].

The equivalent electric circuit in Figure 6c is proposed to fit the EIS data. The fitted parameters are listed in Table 2. *R*_s_, *R*_f_, and *R*_ct_ are used to describe the solution resistance, the LDH film resistance, and the charge transfer resistance, respectively [60]. *CPE*_f_ and *CPE*_dl_ represent the non-ideal capacitance behaviors caused by the existence of the LDH film capacitance and the electric double layer capacitance, respectively. With the anion exchange reaction times, the *R*_f_ values remarkably improve, illustrating that the thickness of the LDH–8HQ films gradually increases [54]. Therefore, the existence of dense film effectively impedes the corrosion reactions, which is in good agreement with the previous analysis (Figure 1 and Figure 2). Generally, a higher *R*_ct_ value indicates better corrosion resistance of the film [49]. The 6 h of 8HQ-loaded LDH film exhibits the highest *R*_ct_, which suggests it behaves with excellent corrosion resistance. It is because chloride anions in a corrosive environment can be replaced into the LDH film through an anion exchange process. Hence, reducing the concentration of chloride anions can effectively weaken the penetration and damage of the active chloride anions to the substrate [61].

### 3.3. Effect of 8HQ on the Long-Term Corrosion Resistance of the LDH Films

In order to deeply explore the corrosion protection capability of the LDH films, the 6 h of 8HQ-loaded LDH film—because of the most promising corrosion inhibition and good surface morphology among the different films—was selected to be treated in 3.5 wt.% NaCl solution for long-term corrosion resistance test.

**Figure 7:**
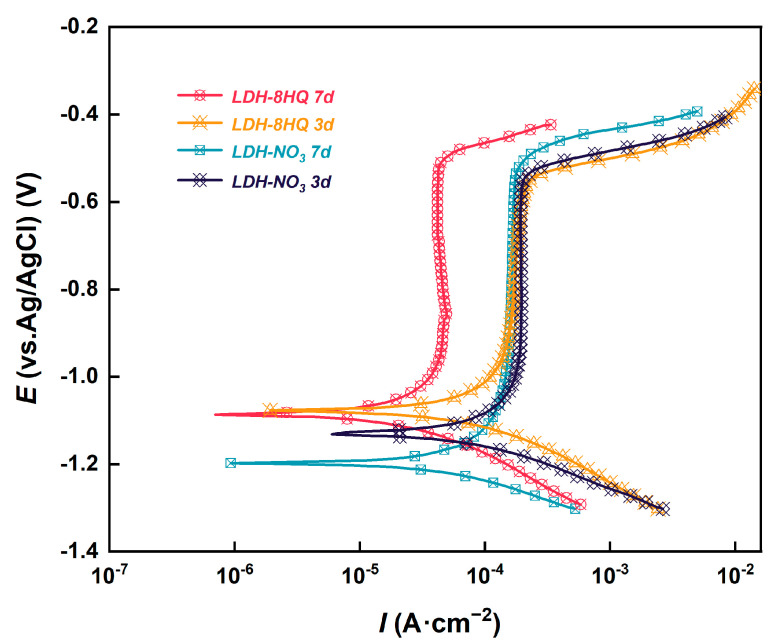
Potentiodynamic polarization curves of the samples of immersion in 3.5 wt.% NaCl solution for 3 and 7 d.

Figure 7 depicts the potentiodynamic polarization curves of the LDH films before and after loading 8HQ anions, and after immersion in 3.5 wt.% NaCl solution for 3 and 7 days. The corresponding electrochemical parameters are presented in Table 3. There is a significant reduction in *I*_corr_ and an obvious shift of *E*_corr_ towards more noble values after immersion for 3 d of the LDH-NO_3_ film. Then, the *I*_corr_ further reduces to 1.008 × 10^−4^ A·cm^−2^ after 7 d. The intercalated NO_3_^−^ anions in LDH layer entrap chloride anions by anion exchange process with immersion time, which enhances the corrosion resistance of AA2024 [57,62]. For the LDH–8HQ film, the *E*_corr_ shifts positively from −1.095 to −1.076 V, while the *I*_corr_ reduces from 1.511 × 10^−4^ to 1.179 × 10^−4^ A·cm^−2^ after immersion for 3 d. Excitingly, the lowest *I*_corr_ (5.586 × 10^−5^ A·cm^−2^) is achieved after further immersion to 7 d. The *I*_corr_ of the LDH–8HQ film is approximately one order of magnitude lower than those of the LDH–NO_3_ film, indicating that it has a superior self-healing ability in the corrosion evolution process. The significant improvement in corrosion inhibition is demonstrated by the remarkable decrease in *I*_corr_ and the obvious positive shift in *E*_corr_ after the LDH loading with 8HQ. Through the anion exchange between 8HQ and chloride anions, chloride anions are entrapped into the LDH galleries [8,63]. The *b*_a_ values move to higher values and the *b*_c_ values move to lower values in the presence of 8HQ [27]. Both anodic and cathodic reactions are suppressed during the inhibitor intercalation, which suggests that the 8HQ reduces anodic dissolution and also retards the oxygen reduction reaction [64,65].

**Figure 8:**
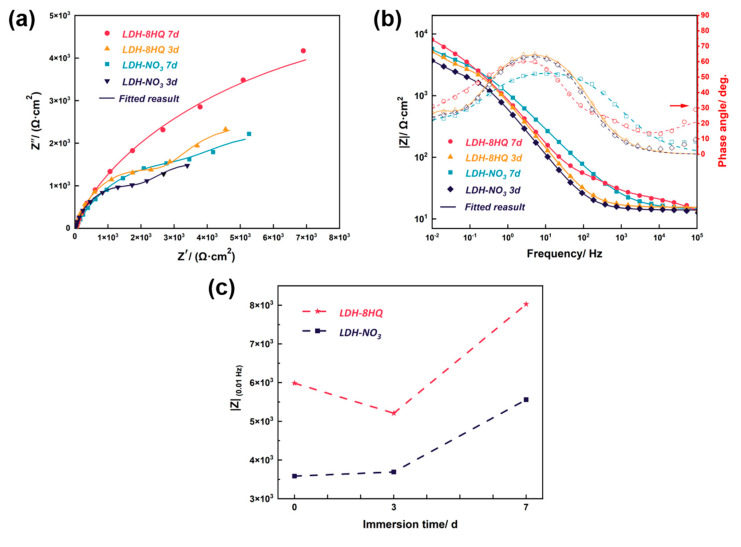
(**a**) Nyquist plots, (**b**) Bode plots and (**c**) |Z|_0.01 Hz_ values as a function of the immersion time of the samples immersed in 3.5 wt.% NaCl solution for 3 and 7 d.

Figure 8 displays the EIS curves of the samples with exposure in 3.5 wt.% NaCl solution for 3 and 7 days, before and after the 8HQ are intercalated into the LDH-NO_3_ film, respectively. Both the Nyquist and the Bode impedance plots are presented in Figure 8a,b, respectively.

A larger capacitive circuit radius points to higher corrosion resistance [66,67]. The LDH–8HQ film possesses the largest capacitive circuit radius in the Nyquist plots in Figure 8a even after immersion to 7 d, signifying the inhibitor molecules are adsorbed on AA2024 surface [68]. In the Bode plots of the phase angle in Figure 8b, the phase angle of the LDH-NO_3_ film is close to 0°, implying that its protective ability gradually diminishes after 7 d of immersion, accompanied by a decrease in the electrochemical response of the barrier layer [61]. Meanwhile, the phase angle of the LDH–8HQ film in high frequency is larger than that of other films, which proves that it has protective film on aluminum alloy after 7 d [69,70].

The impedance modulus at low frequency (|Z| at 0.01 Hz) of the LDH films before and after loading 8HQ were collected at different immersion times (x = 0, 3, and 7 d), as shown in Figure 8c. The |Z| values for the LDH–NO_3_ film continually increase with the immersion time. This phenomenon benefits from the special structure and anion exchange property of the LDH. The LDH–NO_3_ film improves the corrosion resistance of aluminum alloy by capturing corrosive chloride anions and releasing nitrate ions existing between the inter-layer [71]. The LDH–8HQ film keeps the maximum |Z| value after 7 d of immersion, showing the positive impact for the existence of 8HQ on the corrosion inhibition process. Furthermore, the self-healing effect of the film enhances its ability to protect against corrosion [72,73]. The same EIS behaviors are observed for the samples after long-term immersion. The electrochemical parameters are obtained by fitting the EIS data with the described equivalent electric circuit and the findings are summarized in Table 4. The rise in *R*_ct_ values, as reported in previous studies [27,74,75], is ascribed to the augmentation in resistance and the adsorption of inhibitor molecules on the surface of aluminum alloy. This suggests that the film has the ability to self-heal any defects that may occur during the corrosion process [43,52].

### 3.4. Effect of 8HQ on the Fluorescence Performance of the LDH Films

**Figure 9:**
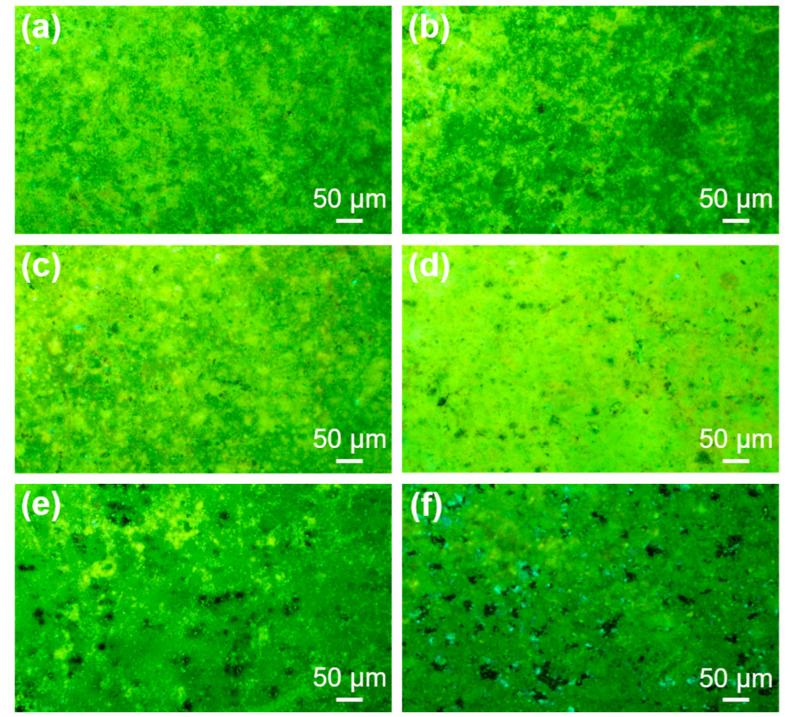
Fluorescence micrographs of the samples after immersion in 3.5 wt.% NaCl solution for different times: (**a**) 0 d, (**b**) 3 d, (**c**) 7 d, (**d**) 10 d, (**e**) 14 d and (**f**) 21 d.

Based on the above morphology, structure, and corrosion resistance, the LDH film loaded with 8HQ for 6 h was chosen and immersed in 3.5 wt.% NaCl solution for different times (x = 3, 7, 10, 14 and 21 days) to study the fluorescence intensity of the film and further to evaluate the corrosion evolution on the substrate. Figure 9 exhibits the fluorescence micrographs of the samples after immersion in 3.5 wt.% NaCl solution for different times. It is found that the AlQ_3_ produced by the chelation of 8HQ and Al^3+^ has a certain fluorescence frequency and wavelength under ultraviolet excitation [23].

In this experiment, the 8HQ anions complex with Al3+ in the LDH film to turn on fluorescence. As shown in Figure 9a, the fluorescence micrograph of the LDH–8HQ film before immersion can clearly show that the entire film surface emits uniform blue-green fluorescence. It is verified that the LDH–8HQ film was successfully synthesized on AA2024 surface, providing an effective barrier for the substrate. When immersed in 3.5 wt.% NaCl solution, due to the anion exchange between 8HQ and chloride anions, chloride anions are trapped in the LDH galleries and 8HQ anions are released to the film surface. At this time, both anode and cathode reactions are inhibited, indicating that the LDH film has a better self-healing ability in the process of corrosion evolution. The samples immersed for 3, 7, and 10 d show few corrosion points in Figure 9b–d. More chloride anions are trapped in the LDH galleries with the immersion time, resulting in “turn-off” fluorescence. After immersion for 14 or even 21 d in Figure 9e,f, the fluorescence quenching points on the surface are more and more, and the areas of fluorescence quenching points are further expanded, indicating that the corrosion of the film is intensified. By virtue of the anion exchange characteristics of the LDH film and the photosensitive compound 8HQ, the accurate location of the corrosion area and the amplification of the corrosion signal are realized, which is conducive to more intuitive and obvious monitoring and warning of the corrosion occurrence.

### 3.5. The Mechanism for “Smart” LDH Films with Sensor and Protection Dual Functions

The LDH–NO_3_ film was synthesized on aluminum alloy AA2024 by the hydrothermal process. Due to the compact oriented growth of the film, it possesses the capability of passively blocking corrosive ions. The reduction of corrosion ions concentration can effectively weaken the penetration and damage of active corrosive ions to the substrate. The LDH–8HQ film was prepared on the basis of the LDH-NO_3_ film using anion exchange reaction. On the one hand, Cl^−^ anions are trapped in the LDH galleries on account of the anion exchange between 8HQ and corrosive Cl^−^ to prevent it from further diffusing to the substrate in 3.5 wt.% NaCl solution. On the other hand, 8HQ are released to the surface for adsorption. As the green and sustainable mixed corrosion inhibitor, 8HQ functions by blocking the active sites on the metal surface to prevent the corrosion of aluminum alloy. This inhibition not only impedes the anodic dissolution reaction, but also hinders the cathodic oxygen reduction reaction.

**Figure 10:**
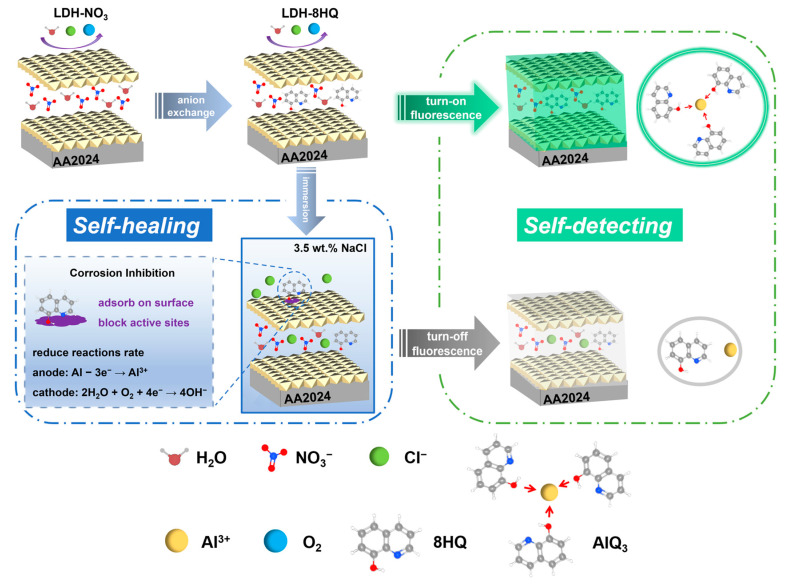
Mechanism diagram of “smart” LDH films with sensor and protection dual functions.

Simultaneously, 8HQ in the LDH-8HQ film inter-layer can combine with Al^3+^ in the LDH laminates to turn on fluorescence under ultraviolet radiation. After immersion in 3.5 wt.% NaCl solution, 8HQ exchange with Cl^−^ anions and lose their complexation with Al^3+^ in the laminates, thus turning off the fluorescence. The mechanism for “smart” LDH films with sensor and protection dual functions is shown in Figure 10.

## 4. Conclusions

In conclusion, the LDH film loaded with 8HQ has been proved to be used for the corrosion self-healing and self-detecting of aluminum alloy. The LDH–NO_3_ film was in-situ grown on aluminum alloy surface, and the LDH film loaded with 8HQ anions for 6 h was prepared by anion exchange property. The macroscopic morphologies show that the color changed from white to yellow evenly. The micromorphologies have no obvious change, and the LDH nanosheets are uniformly and densely covered on AA2024 surface. The LDH-8HQ film thickness is increased to about 6 μm, and tightly combined with the substrate. The results of potentiodynamic polarization curves and electrochemical impedance spectroscopy (EIS) indicate that the LDH film loaded with 8HQ for 6 h improves the corrosion resistance of the matrix. The long-term corrosion resistance test manifests that the adsorption of 8HQ can significantly inhibit the anodic and cathodic reactions and can be used as an efficient corrosion inhibitor for aluminum alloy in 3.5 wt.% NaCl solution. The photosensitive compound 8HQ can interact with Al^3+^, showing obvious fluorescence enhancement or weakening behaviors. It has a self-detecting ability to convey film damage or performance degradation in the form of fluorescent signal (color change). During immersion for 21 d in corrosive solution, the LDH–8HQ film changes from fluorescence enhancement to fluorescence quenching.

The 8HQ-loaded LDH film has proven to be the successful strategy of corrosion sensor and inhibitor for aluminum alloy. The quick release of 8HQ from the LDH film triggers “turn-off” fluorescence and inhibits corrosion to restore the film protection. The “smart” LDH films with sensor and protection dual functions provide promising opportunities to meet the autonomous life-cycle control of materials and to reduce the risk of accidents.

## Figures and Tables

**Table 1:** Parameters of potentiodynamic polarization curves for the samples immersed in 3.5 wt.% NaCl solution.

Sample	*E*_corr_ (V)	*I*_corr_ (A·cm^−2^)	*b*_a_ (mV·dec^−1^)	*b*_c_ (mV·dec^−1^)	*R*_p_ (Ohms/cm^2^)	*η* (%)
Blank	−1.225	9.532 × 10^−4^	294.5	−102.6	3.466 × 10^4^	/
LDH-NO_3_	−1.161	8.945 × 10^−4^	387.4	−110.3	4.167 × 10^5^	91.7
LDH-8HQ 5 min	−1.126	1.529 × 10^−4^	1528.1	−165.7	4.246 × 10^5^	91.8
LDH-8HQ 30 min	−1.127	1.430 × 10^−4^	1466.1	−148.3	4.089 × 10^5^	91.5
LDH-8HQ 1 h	−1.157	1.113 × 10^−4^	895.6	−132.2	4.494 × 10^5^	92.3
LDH-8HQ 6 h	−1.095	1.511 × 10^−4^	3373	−210.5	5.692 × 10^5^	93.9

**Table 2:** EIS fitted parameters of the samples immersed in 3.5 wt.% NaCl solution.

Sample	LDH-NO_3_	LDH-8HQ 5 min	LDH-8HQ 30 min	LDH-8HQ 1 h	LDH-8HQ 6 h
*R*_s_	15.45	28.08	13.95	15.21	15.18
*CPE*_f_	3.54 × 10^−4^	3.39 × 10^−4^	5.04 × 10^−4^	3.16 × 10^−4^	1.37 × 10^−4^
*α*_f_	0.73	0.89	0.83	0.74	0.87
*R*_f_	3098	1125	1115	1592	4898
*CPE*_dl_	9.63 × 10^−3^	1.15 × 10^−3^	9.29 × 10^−4^	5.42 × 10^−4^	1.03 × 10^−2^
*α*_dl_	1.06	0.65	0.59	0.41	1.18
*R*_ct_	1006	3738	4372	4432	4651

**Table 3:** Parameters of potentiodynamic polarization curves for the samples immersed in 3.5 wt.% NaCl solution for 3 and 7 d.

Sample	*E*_corr_ (V)	*I*_corr_ (A·cm^−2^)	*b*_a_ (mV·dec^−1^)	*b*_c_ (mV·dec^−1^)	*R*_p_ (Ohms/cm^2^)	*η* (%)
Blank	−1.225	9.532 × 10^−4^	294.5	−102.6	3.466 × 10^4^	/
LDH-NO_3_ 3 d	−1.130	1.569 × 10^−4^	1250.3	−148.1	4.664 × 10^5^	92.6
LDH-NO_3_ 7 d	−1.198	1.008 × 10^−4^	898.38	−141.3	5.258 × 10^5^	93.4
LDH-8HQ 3 d	−1.076	1.179 × 10^−4^	823.97	−170.1	5.195 × 10^5^	93.3
LDH-8HQ 7 d	−1.086	5.586 × 10^−5^	9240.1	−201.3	1.531 × 10^6^	97.7

**Table 4:** EIS fitted parameters of the samples immersed in 3.5 wt.% NaCl solution for 3 and 7 d.

Sample	LDH-NO_3_ 3 d	LDH-NO_3_ 7 d	LDH-8HQ 3 d	LDH-8HQ 7 d
*R*_s_	14.17	13.82	15.46	13.95
*CPE*_f_	2.47 × 10^−4^	2.54 × 10^−4^	2.73 × 10^−4^	3.54 × 10^−4^
*α*_f_	0.64	0.64	0.79	0.79
*R*_f_	4883	3968	3548	2510
*CPE*_dl_	2.73 × 10^−3^	2.95 × 10^−3^	3.29 × 10^−3^	3.66 × 10^−3^
*α*_dl_	0.79	0.84	0.92	0.82
*R*_ct_	4282	4371	4369	5179

## Data Availability

Not applicable.
